# Homicide and Associated Steroid Acute Psychosis: A Case Report

**DOI:** 10.1155/2011/564521

**Published:** 2011-11-28

**Authors:** G. Airagnes, C. Rouge-Maillart, J.-B. Garre, B. Gohier

**Affiliations:** ^1^Service de Médecine Légale, CHU d'Angers, 49933 Angers Cedex 09, France; ^2^Département de Psychiatrie et de Psychologie médicale, CHU d'Angers, 49933 Angers Cedex 09, France; ^3^IFR 132, Université d'Angers, 49035 Angers, France

## Abstract

We report the case of an old man treated with methylprednisolone for chronic lymphoid leukemia. After two months of treatment, he declared an acute steroid psychosis and beat his wife to death. Steroids were stopped and the psychotic symptoms subsided, but his condition declined very quickly. The clinical course was complicated by a major depressive disorder with suicidal ideas, due to the steroid stoppage, the leukemia progressed, and by a sudden onset of a fatal pulmonary embolism. This clinical case highlights the importance of early detection of steroid psychosis and proposes, should treatment not be stopped, a strategy of dose reduction combined with a mood stabilizer or antipsychotic treatment. In addition have been revised the risks of the adverse psychiatric effects of steroids.

## 1. Introduction

Argentine physiologist Bernardo Houssay identified cortisone and described its main actions on the body at the beginning of the 20th century. In 1950, the three researchers Philip Hench, Edward Kendall, and Tadeus Reichstein received the Nobel Prize for physiology and medicine for “the isolation of cortisone and the introduction of its clinical use” and corticosteroids were commercialized. Within a year, initial reports were received of their undesirable psychiatric effects [1, 2]. 

Cortisone is secreted by the adrenal cortex, under the control of the hypothalamus, which is itself regulated by the limbic system (tonsils, hippocampus) and the anterior pituitary gland. The increase in the secretion of glucocorticoids follows an acute event deemed as stressful by the limbic system (mobilization of energetic resources and inhibition of auxiliary activities) [3].

Corticosteroid therapy is today a treatment which has many broad uses, from autoimmune and system diseases to severe inflammatory and allergic reactions, as well as in palliative care and support [2, 4]. The counterweight of these broad therapeutic indications is witnessed in numerous undesirable side effects, including amongst others metabolic, endocrine, digestive, immunological, ophthalmological, dermatological, and neuropsychiatric disorders. Concerning corticosteroid-induced adverse psychiatric effects, the literature remains undeveloped from a pharmacoepidemiological perspective and recent reviews principally consist on summarizing published data based on case reports and case series [5, 6]. However, links between HPA axis dysfunction and psychosis, mood, or anxiety disorders were clearly established [7]. An impairment in glucocorticoid receptors function (glucocorticoid resistance) could be responsible of their “depressogenic” effect in patients with increased levels of glucocorticoids like in Cushing's syndrome or in exogenous treatment with synthetic glucocorticoids [8, 9]. Corticosteroids are associated with changes in the temporal lobe, detected by structural, functional, and spectroscopic imaging [10]. As regards the undesirable neuropsychiatric effects, these affect around 34% of patients with 6% of patients experiencing severe reactions. Dosage is directly related to the incidence of adverse effects but is not related to the timing, severity, or duration of these effects Corticosteroid-induced symptoms frequently occur during the first few weeks of therapy and typically resolve with dosage reduction or discontinuation of corticosteroids [11]. No specific personality has been identified as a risk factor for corticosteroid-induced psychosis episode. Moreover, it is not possible to determine whether past psychiatry illness disorder could be risk factors for the development of a corticosteroid-induced psychiatric episode [12].

We report on the case of a patient who presented an acute corticosteroid-induced psychotic reaction, resulting in the committing of a heteroaggressive deed which led to the death of his spouse.

## 2. Case Report 

We reported the case of a 77-year-old man, living with his wife, who had four children: three daughters and a son. He had never suffered from any previous psychiatric illness. In April 2009, he was treated with pregabalin (450 mg/day) and codeine (120 mg/day) shingles. At this moment, no biological signs of immunodepression were found. After 15 days, he suddenly presented impairments in temporal and attentional orientation, deficits in consciousness and memory, suggesting a confusional state [13]. The treatment was stopped because of adverse effects. 

In June 2009, the patient complained on an important fatigue and his general practitioner (GP) prescribed a blood cells count, which was found around 28000/*μ*L, with a monoclonal proliferation of B cells. The chest radiography highlighted a mediastinal adenopathy. The cerebral scanning was normal, and the infectious blood tests were negative for HIV and treponema. In July 2009, an internal physician concluded to a chronic lymphoid leukemia and purposed a treatment using methylprednisolone (80 mg/day), and this obtained a good response. After 22 days of treatment, the blood count was normalized. However, the patient complained of sleep disorders, such as sleep discontinuation, early awakenings, temporal and spatial disorientation, and mood dysregulation (euphoria). The first of September, when his neoplasia was stabilized, he called himself emergency services and told them that “he made a mistake.” At home, the rescuers found his wife laid down on the floor, with several injuries, beaten to death. The patient, dazed, walked around his wife. He spoke to himself with incomprehensible words. It was impossible to communicate with him. This disorganization, both the emotional state and behavior or language, suggests psychotic symptoms. The autopsy of the wife revealed: broken nose, broken right elbow, broken left humerus, broken ribs, meningeal hemorrhage, bilateral hemothorax, pulmonary contusions, with much bruising and many contusions to the face, arms, and legs. 

The emergency services diagnosed an acute delirious state (poisoning ideas, visual hallucinations), a dissociative syndrome, and elements of confusion such as amnesia, disorientation, and symptomatological fluctuation. He needed attention and was hospitalized without his consent. He was disorientated and unable to remember what had happened. For him, it was a contrivance, and he thought that his wife was still alive. His reaction was very anxious and nervous. Hyponatremia (126 mmol/L) and community pneumonia (by *streptococcus pneumoniae*) were also found. The brain scanning was normal. His family reported potomania's tendencies that could explain the hyponatremia. He took no treatment susceptible to interact with ADH. The steroids were stopped, and delusional symptoms declined in 72 hours, after a treatment by loxapine (100 mg, once daily). Natremia was progressively corrected by serum [Na+]. He realized what he had done and decided to refuse to live. He felt feelings of guilt and shame. He progressed to a major depressive disorder with suicidal ideas. Following steroid stoppage, the leukemia progressed. 

On the 11th of September, he presented respiratory insufficiency and died consequently to a pulmonary embolism on the 21st of September. An autopsy should be interesting but was not asked by the judge. According to Naranjo's algorithm, the relationship between corticosteroid and adverse effects could be possible (total score +3) [14].

## 3. Discussion

This case is an example of the possible risk of iatrogenic psychotic reactions in the occurrence of forensic acts. Several arguments could be raised to support the link between taken corticosteroids and the murderous behavior.

- The patient already displayed an iatrogenic reaction in April 2009 following the implementation of treatment using pregabalin and codeine-based paracetamol for shingles in the lower back. His state of secondary confused agitation then rapidly declined following stoppage of treatment. As a preexisting neurological disorder, we might suppose a sensibility for this patient to iatrogenic effects, but it is not sufficient to clearly establish a relationship between corticosteroids and forensic behaviour.
- Previously, he never suffered from psychiatric symptoms requiring psychiatric consultation, hospitalization, or specific drugs.
- The symptoms were fluctuating, appearing rapidly after the start of treatment, composed of an acute delusional syndrome with episodes of agitation and disorientation linked to memory difficulties, attention deficit, and an inversion of the day/night cycle. No signs of dementia were found before the murder, in particular by his GP.
- Significant sleep disorder without fatigue presented just after the start of methylprednisolone were most likely already signs of a corticosteroid-induced psychiatric reaction [15].

The relationship between corticosteroids and murder is not certain in our case, but possible. Other factors are well known to lead to a confusional syndrome such as hyponatremia and infectious state. As regards the sepsis, we know that it appears in an atypical way in the form of a confused syndrome in 52% of elderly subjects [16]. In our case, it could be advanced that the pneumonia, due to the immunodepressed state, which is itself due to the chronic lymphoid leukemia, added to the hyponatremia, contributed to weakening cerebral homeostasis, and thus facilitated the occurrence of iatrogenic effects, such as cortico-induced psychosis.

That is why it is important to detect early steroid psychosis and to propose, should treatment not be stopped, a strategy of dose reduction combined with a mood stabilizer or antipsychotic treatment, for example, haloperidol or chlorpromazine. Olanzapine and risperidone appear limited to subacute cases. Theoretically, lithium could be used as prophylaxis for corticosteroid-induced psychosis with beneficial effects. But, in clinical practice, corticosteroids-induced changes in sodium balance might increase the risk of lithium intoxication and renal dysfunction. Other mood stabilizers have been tested like carbamazepine, which decrease serum concentration of prednisolone, or valproate, but they are not sufficiently documented to be recommended. A decline in declarative and working memory is also reported during corticosteroid therapy. Lamotrigine and memantine have been shown to reverse, at least partially, the declarative memory effects of corticosteroids [10]. Some studies reported beneficial effects of serotonin reuptake inhibitor but also less documented. Dosage had to be ascertained case by case. Speed of improvement is very inconstant. However, most of the patients improved in the first three days with a tail-off period to bring recovery. Disease management strategies are insufficiently documented to be recommended [17–19].

Reckart and Eisendrath showed that over 80% of patients undergoing corticosteroid therapy recruited to their study had not been informed of the risks of the occurrence of psychiatric side effects, while 50% of patients complained of various symptoms (insomnia, euphoria, confusion, memory difficulties, depressive symptoms) and, in 62% of cases, they had not informed the prescribing party of the occurrence of these side effects [20]. It seems difficult to bridge the gap in terms of information about the psychiatric side effects of corticosteroids, due both to their frequency and their potential seriousness, as this clinical situation shows. Information about common side effects such as a change in mood must be systematically given to the patient and those around him/her. It may be supposed that once informed of these possible iatrogenic effects, the patient will pay more attention and report any symptoms early should they occur, which may in some situations prevent an evolution into more serious difficulties, such as corticosteroid-induced psychosis. Moreover, any physician must have specific relationships also with caregivers who may be their first to become aware of the adverse psychological effects of the drug. 

## 4. Conclusion

This clinical illustration underlines the importance of bearing in mind the possible occurrence of serious psychiatric side effects due to corticosteroids, even if these are exceptional in nature. The extremely acute onset of symptoms, the onset of delusions, a history of neuropsychiatric iatrogenic disorder, the existence of somatic precipitating disorders, and confusion factors should always alert clinicians to said possibility. The patient and those surrounding him/her must be made aware of the risks of adverse psychiatric side effects of steroids, for both ethical and forensic reasons, and these should be reported as soon as possible to the clinician should they occur.
